# Dental Glass Ionomer Cements as Permanent Filling Materials? —Properties, Limitations Future Trends

**DOI:** 10.3390/ma3010076

**Published:** 2009-12-28

**Authors:** Ulrich Lohbauer

**Affiliations:** Dental Materials Lab, Dental Clinic 1, University of Erlangen-Nuremberg, Glueckstrasse 11, 91054 Erlangen, Germany; E-Mail: lohbauer@dent.uni-erlangen.de; Tel.: +49 9131 8543740; Fax: +49 9131 8533603

**Keywords:** glass ionomer cement, strength, wear, fatigue, brittleness, porosity, glass fibers, resin modification, coating, clinical, restoration

## Abstract

Glass ionomer cements (GICs) are clinically attractive dental materials that have certain unique properties that make them useful as restorative and luting materials. This includes adhesion to moist tooth structures and base metals, anticariogenic properties due to release of fluoride, thermal compatibility with tooth enamel, biocompatibility and low toxicity. The use of GICs in a mechanically loaded situation, however, has been hampered by their low mechanical performance. Poor mechanical properties, such as low fracture strength, toughness and wear, limit their extensive use in dentistry as a filling material in stress-bearing applications. In the posterior dental region, glass ionomer cements are mostly used as a temporary filling material. The requirement to strengthen those cements has lead to an ever increasing research effort into reinforcement or strengthening concepts.

## 1. Introduction

During the last decades, an increasing variety of dental restorative materials have conquered the market. Gold and ceramics are the main standard material used for indirect restorations, and until the late seventies amalgam was used for direct restorations [1]. The use of amalgam has been critically discussed due to its allergic and toxic potential upon mercury release [2]. Today, the decreased number of amalgam fillings is also influenced by a high demand for tooth-colored and biocompatible restorations [1]. Great strides in dental research have led to a variety of alternatives to amalgam [3]. An increased demand for direct filling materials was supported by changes in restorative techniques. The development of adhesive techniques saves sound tooth structure and is compatible with prophylactic concepts. Preserving and stabilizing tooth hard tissues by direct filling techniques is more and more in favor, in contrast to macromechanically styled, destructive preparations with indirect restorative materials [4].

Different types of direct restorative materials are used in daily dental practice. The most common, next to amalgam, are resin composites, and glass-ionomer cements (GICs). Amalgam, with its long clinical history, is inexpensive and easy to handle. However, the possible toxicity caused by mercury and poor esthetics are disadvantages [2]. Resin composites are the most esthetically accepted material with satisfactory physical properties [5]. They show their drawbacks in a being a highly expensive, time-consuming and technique-sensitive adhesive procedure [6,7]. Glass-ionomer cements may be used in a wide range of clinical applications due to the ability to modify their physical properties by changing the powder/liquid ratio or chemical formulation [8]. The glass-ionomer cements are esthetically more attractive than metallic restorations [9]. In addition, by incorporating fluorine, they exhibit an anticariogenic potential, and they have good biocompatibility and chemical adhesion to mineralized tissue [10]. On the other hand, poor mechanical properties, such as low fracture strength, toughness and wear, limit their extensive use in dentistry as a filling material in stress-bearing areas [11,12]. In the posterior dental region, glass-ionomer cements are mostly used as a temporary filling material [13]. The requirement to strengthen those cements has lead to an increasing research effort into reinforcement concepts. Several former approaches dealt with incorporation of second phase ceramic or glass fibers or with metal particles [14]. Encouraging results were also obtained by compounding reactive glass fibers [15,16].

## 2. Historical Development

The development of amalgam, gold and porcelain restorative materials in the first half of the 19th century stimulated the development of dental cements as luting and lining materials and as more esthetic restorative materials. By the end of the first quarter of the 20th century three basic types of cements: zinc oxide eugenol (1875), zinc phosphate (1879) and silicate cement (1908) were established for the bonding of inlays, crowns, posts, bridges and orthodontic bands onto or within the tooth and as cavity linings, bases and filling material [7,17].

In the early sixties it became evident that hydrophilic materials capable of wetting and reacting with hydroxyapatite (HA) and/or the collagenous phase of tooth tissue (dentin) were required for durable bonding to the tooth structure. Due to the presence of HA in both enamel and dentin, reactants that chelate or complex to calcium seemed most promising. At this time there was a growing interest in water soluble polyelectrolyte systems containing citric and polycarboxylic acids. In 1963, the potential of polyacrylic acid to adhere to dental tissue was investigated for the first time. This adhesive quality was due to the ability of polyacrylic acids to complex with calcium and the formation of hydrogen bonds with organic polymers comparable to collagen [18]. As a result, materials containing fillers, fluorides and copolymers such as polycarboxylic acid became commercially available. In addition to their biocompatibility and good physical properties, such as high compressive strength, the major new feature of these polyacrylate cements was their ion-binding potential to the hydroxyapatite phase of dentin and enamel [7]. Wilson and Kent [19] developed silicate cements with improved esthetics by modification of the Al_2_O_3_/SiO_2_ ratio in the silicate glass. High fluorine-containing glasses were found to react with polycarboxylic acids and, by employing the key effect of tartaric acid on setting properties, the first practical glass-ionomer cement (ASPA) was introduced to the market in 1972 [14]. The evolution of the GIC over the last decades has resulted in changes in both the glass powder component and the polycarboxylic acid. In this period, clinical experience has highlighted the practical advantages and disadvantages of the GIC system. The principles of today’s GIC are well understood, which in turn has led to improved formulations and highly reproducable techniques [20]. However, the main problem of a weak strength and toughness for permanent filling therapy still remains.

## 3. Cement Composition

### 3.1. Acid-Base Reactions

**Figure 1:**
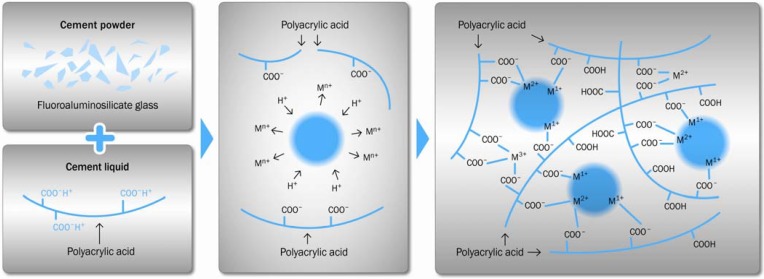
Setting reaction of a conventional glass-ionomer cement.

Dissolution of inorganic glasses by acid solution is normally undesirable. However, with ionomer glasses the glass composition is designed to be degradable by relatively weak acids in order to form a cement. Typically an aqueous polyacid, such as polyacrylic acid, is reacted with the finely powdered fluoroaluminosilicate glass [20]. This acid-base setting reaction is schematically shown in Figure 1.

The acid attacks the glass network which results in the release of cations, mainly Al^3+^ and Ca^2+^ or Sr^2+^. The cations subsequently serve to form salt bridges between the polyacid chains and result in the formation of silica hydrogel, the calcium polyacrylate formation exhibiting faster reaction kinetics than that of aluminum polyacrylate [21].

The carboxylic acid attacks the surface layer of glass powder, whereas the glass core remains intact. The glass core acts as filler in the cement matrix. The reactivity of the glass surface determines the quality of the set cement. A silica gel layer with gradient properties is formed at the interface between the glass particle and the cement matrix. The polycarboxylic acid is typically used in aqueous solution at a concentration of 45 wt %. In order to control the GIC setting kinetics, a certain amount of dried polycarboxylic acid is added to the glass powder.

**Figure 2:**
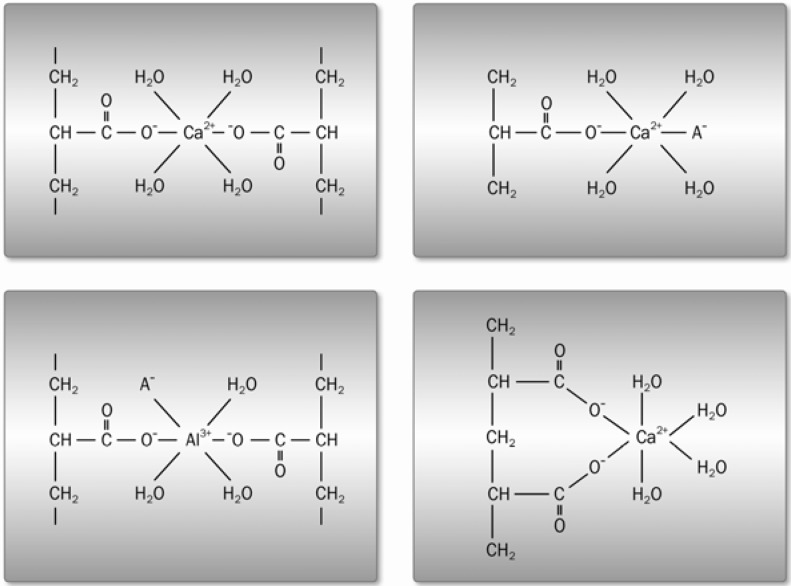
Principal hydrolysis configurations during cement maturation [7].

**Figure 3:**
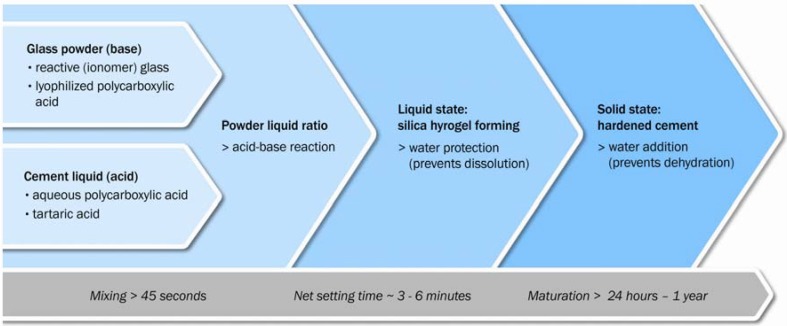
Principal setting stages during cement maturation.

Water plays a critical role in the setting process. During the first stages of the setting process, the water from the cement liquid is fully incorporated into the cement structure [22]. During cement setting, the cement paste has to be protected from additional water in order to prevent from dissolution of metal cations. Once the cement has set into a solid state, water can occupy various locations, for example coordination sites around metal cations or hydration regions around the polyanion chain [23,24]. The principal of water uptake in GIC during maturation can be seen in Figure 2. At this stage, loss of water can lead to cracking and crazing of the cement surface, resulting in a chalky surface appearance [25]. As the cement ages the proportion of loosely bound water decreases relatively to the proportion of tightly bound water. The setting process is observed to continue with time [26,27]. The systematic cement setting stages are summarized in Figure 3.

### 3.2. Reactive (Ionomer) Glasses

The glasses used in these cements have a complex structure and consist of many components. However, the three principal components are silica (SiO_2_), alumina (Al_2_O_3_), and lime (CaO). CaO is often substituted by strontium (SrO) or zinc (ZnO) oxides. Fluorite (CaF_2_) is also incorporated as a source for fluoride release. In addition, they often contain phosphate (P_2_O_5_) and soda (Na_2_O) [14]. Such compositions are similar to commercial Bioglass^®^ (University of Florida, Gainesville, FL) [28]. Wilson *et al.* [20] studied the relationship between the initial glass composition and the properties of the resulting cement pastes. The most important factor in determining cement properties is the Al:Si ratio in the glass [29]. However, this ratio cannot be considered in isolation since the mole fraction of network-modifying cations, such as Ca^2+^ or Sr^2+^, largely determines the structural role of aluminum within the glass network [30]. If the Ca:Al mole ratio > 1:2 and the Al:Si ratio < 1:1, then all the aluminum is in fourfold coordination and the aluminum may enter the glass network as [AlO4] tetrahedron. The glass network then consists of linked [ALO_4_] and [SiO_4_] tetrahedrons. In such a structure, when Al^3+^ replaces Si^4+^, the glass network glass network forming units have a negative charge, which is balanced by the positive charge of a network-dwelling cation [31]. Should there be insufficient network-dwelling cations, *i.e.,* the Ca:Al ratio < 1:2 then not all of the aluminum ions can adopt fourfold coordination and some take up sixfold coordination. The resulting oxygen linkage between adjacent aluminum and silicon tetrahedrons is vulnerable to acid attack. The Al^3+^ ion has a weaker field strength than that of the Si^4+^ ion [32]. As a result, the Al^3+^ ion interacts less strongly with the electron clouds of the oxygen anions, leaving them with sufficient residual polarizability to be susceptible to acid attack. Non-bridging oxygens are similarly susceptible to acid attack—the counterbalancing cations are exchanged for protons. Fluorides such as CaF_2_ are introduced into these glasses in order to form [AlO_3_F] and [SiO_3_F] tetrahedrons [32,33]. The replacement of O_2_^-^ ions by F^-^ ions reduces the screening of the central cation and so strengthens the remaining cation bonds, making them less susceptible to acid attack. However, the F^-^ ion is non-bridging and therefore disrupts the glass network [32,34]. Effectively, every fluorine ion introduces a non-bridging oxygen ion into the glass network. Thus, CaF_2_ is in principle a much more powerful network modifier than CaO, and indeed the replacement of CaO by CaF_2_ increases the susceptibility of a glass towards acid attack and reduces the setting time of the GIC pastes [35]. Work from Wood and Hill [36] on some multi-component glasses showed that, in addition to crystallization, many of the GIC glasses also had a liquid-liquid phase separation, to give a droplet phase enriched in calcium and fluorine. In many cases this droplets had subsequently crystallized to CaF_2_. Further studies even showed that the cement reaction occurred preferentially with the calcium and fluorine-rich phase. In general the glasses that had undergone liquid-liquid phase separation gave the highest strength cements [22]. Alkali ions are often added to ionomer glasses to reduce the melting temperature during the manufacturing process and are cited as facilitating fluoride ion release by providing a soluble counter ion [37]. However, incorporation of sodium has a deleterious influence on the solubility, hydrolytic stability and mechanical properties of the cement. Sodium is likely to be released from the glass in greater proportions relative to the other cations present in the glass since sodium is known to be relatively mobile at low temperatures in silicate glasses and can be easily exchanged for hydrogen ions [38]. Sodium ions compete with calcium and aluminum cations for carboxylate groups in the polyacid chains and therefore inhibit the cross-linking process. The extent of cross-linking in the polysalt matrix will influence the Young’s modulus, the extent of plastic deformation at the crack tip and hence the fracture toughness [39].

### 3.3. Polycarboxylic Acids

**Figure 4:**
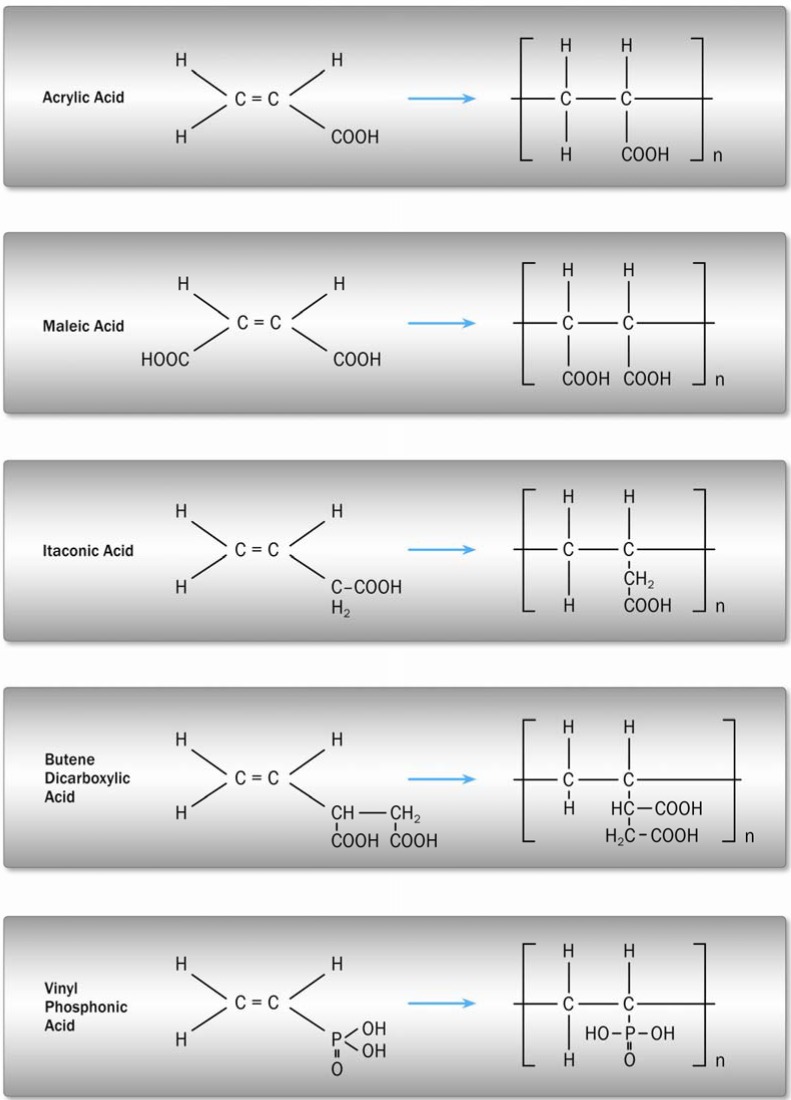
Polycarboxylic acids used for GIC formation [7].

The polyacid that reacts with the ionomer glass is usually a polycarboxylic acid. A variety of glass-ionomer cement forming acids are shown in Figure 4.

The reactivity depends on the ingredients of the acid or of a copolymeric acid, as well as on its molecular weight and concentration. By adding maleic or itaconic acid, the number of carboxylic groups relative to the total molecular weight and thus the reactivity is increased. The polyacid either is part of the liquid as an aqueous solution or is incorporated into the cement powder as a dried powder. In the latter case the liquid is simply water in which the dried polyacid dissolves upon mixing [7,14]. The acid degrades the glass structure, hydrolyses the bonds of the glass network and releases aluminum and calcium cations which are chelated by the carboxylate groups and serve to crosslink the polyacrylic chains [38]. Resulting cement properties are mainly influenced by the degree of crosslinking. It was demonstrated that high crosslinking serves to increasing the Young’s modulus of a set cement [37]. A higher polyacrylic acid concentration will also lower the pH and increase the rate and extent of reaction. Higher acid contents are synonymous with lower water contents. This factor in addition with increased ionic crosslinks results in a lower content of unbound water. It is likely that this will also serve to increase Young’s modulus since unbound water will act as a plasticizer. The toughness properties of GIC were observed to increase with an increased molar mass of the polycarboxylic acid due to an enlarged plastic zone at the crack tip [37]. At very high acid concentrations the reaction may be suppressed by the lack of water for hydrating the complexes formed or by insufficient metal cations being available for a complete neutralization [39].

Adding small amounts of complexing agent will alter the setting behavior of GIC [14]. L-(+)-tartaric acid is the most effective of these additives since it both prolongs working time and provokes a snap set. In the presence of L-(+)-tartaric acid, metal ions are still extracted from the glass, but on release, they apparently react preferentially with the tartaric acid to form the tartrate, and thus delays the formation of the polysalt matrix [26]. L-(+)-tartaric enantiomers not only react rapidly to yield calcium tartrate, but also enhance the rate at which aluminum polyacrylate is formed within the cement [21].

## 4. Material Properties

GIC are clinically attractive dental materials and have certain unique properties that make them useful as restorative and adhesive materials. This includes adhesion to moist tooth structure and base metals, anticariogenic properties due to the release of fluoride, thermal compatibility with tooth enamel, biocompatibility and low toxicity. However, limitations in their applications may result from the low mechanical strength and toughness [40].

### 4.1. Linear-Elastic Mechanical Properties

The basic mechanical parameters characterizing a dental restorative material are their linear-elastic properties upon failure, such as elastic modulus, fracture strength, fracture toughness and surface hardness. Stress-strain characteristics of GIC vary within a broad range due to the applied testing conditions. Commercial products exhibit an elastic modulus of 2–10 MPa [41,42]. Extensive moisture contamination – especially within the first stages after cement mixing – has been attributed as being responsible for reduced elastic modulus and fracture strength [7]. Properties such as their low fracture toughness, mechanical strength and brittleness need to be improved in order to extend clinical indications into the stress-bearing posterior region [43]. *In vitro* flexural or compressive testing has been shown to be appropriate for assessing the mechanical properties close to the clinical loading situation. However, among testing methods, the flexural strength test was considered to produce the most appropriate measure of the material strength which can offer the best practical and reliable estimate of tensile strength [44]. Various researchers have measured the strength of GIC under different conditions. A review of representative data is given in Table 1. The compressive strength of GIC is commonly measured after 24 hours wet storage. Compressive strength ranges between 60 and 300 Mpa and flexural strength up to 50 Mpa (Table 1). GIC exhibit a significant increase (approximately 100%) in flexural as well as in compressive strength when exposed to water in the period between 24 hours and one year after mixing [18,41]. When exposed to aqueous solutions of varying pH, GIC exhibited a high acid erosion resistance compared to other restorative materials [45]. From long-term experiments on GIC, a high water uptake of 5% was measured within the first six months [46]. GIC exhibit a slightly higher volumetric expansion due to this high degree of absorbed water when compared with resin composites [47]. Fracture toughness measurements differ according to the water uptake, since water influences the GIC microstructure. Values between 0.1 and 0.6 Mpam^0.5^ were determined for commercial and experimental products [48,49]. A correlation between polyacrylic acid molecular weight and fracture toughness could not be shown [48]. An in-vitro increase in fracture toughness of 20% was observed following six month storage in water [42].

**Table 1:** Flexural and compressive strength of conventional GIC.

Author	FS [MPa]	CS [MPa]	Material	Test conditions
Bapna *et al.* 2002 [50]	30.8/23.0 47.1/21.4	-	Fuji II	3-PB, as, 24 h/9 m
	17.8/14.6			3-PB, ws, 24 h/9 m
				3-PB, wc, 24 h/9 m
Dowling *et al.* 2009 [51]	-	126/129	Ketac Fil Plus	Capmix, ws, 24 h/Rotomix
		127/131	Fuji II	Capmix, ws, 24 h/Rotomix
		132	Chemfil	Handmix, ws, 24 h
Fleming *et al.* 2003 [52]	-	87.9/67.9	Fuji IX GP	Capsules, ws, 24 h/handmix
		72.7/62.0	Ketac Fil Plus	Capsules, ws, 24 h/handmix
		84.3/68.9	ChemFlex	Capsules, ws, 24 h/handmix
Iazzetti. *et al.* 2001 [53]	22.6/15.4	-	Fuji IX	3-PB, ws, 24 h/7 d
Irie *et al.* 2008 [54]	1.8/29.2	-	Fuji IX GP	3-PB, ws, immediate/24 h
	1.7/17.3		FX-II	3-PB, ws, immediate/24 h
	1.9/19.3		Ketac Molar	3-PB, ws, immediate/24 h
	2.0/15.3		Fuji II	3-PB, ws, immediate/24 h
Lohbauer *et al.* 2003 [41]	19.7/33.0/35.2/36.7	-	Ketac Molar	4-PB, ws, 24 h/8 d/30 d/90 d
Lucksanasombool *et al.* 2002 [49]	29.2	211	Fuji IX	3-PB, ws. 1 h
Moshaverinia *et al.* 2008 [55]	14.8	161.0	Fuji II	BB, ws, 24 h
Peez *et al.* 2006 [56]	51	244	Ketac Molar	3-PB, ws, 24 h
	42	236	Fuji IX	3-PB, ws, 24 h
	48	141	Vitro Molar	3-PB, ws, 24 h
	38	175	Vidrion R	3-PB, ws, 24 h
	36	196	Ionofil Molar	3-PB, ws, 24 h
Prosser *et al.* 1986 [44]	16.4–33.0	-	Experimental	p/l ratio, ws, 24 h
	7.6 – 20.4		Experimental	liquid comp, ws, 24 h
Xie *et al.* 2000 [11]	22.6	251	Ketac Fil	3-PB, ws, 7 d
	21.2	301	Ketac Molar	3-PB, ws, 7 d
	26.1	202	Fuji II	3-PB, ws, 7 d

FS: flexural strength; CS: compressive strength; 3-PB: three-point-bending; 4-PB: four-point-bending; BB: biaxial bending; ws: wet storage; as: air storage; wc: wet cyclic.

### 4.2. Wear and Fatigue

The long term mechanical properties of GIC are generally investigated under simulated oral conditions. The intraoral behavior of restorative materials is a complex process in which masticatory loading in the presence of a chemically active environment accounts for a degradation of the restoration. Over time, the detoriation is described in general terms of wear, marginal breakdown and fatigue fracture due to cyclic loading [57]. Since the level of masticatory force, which impacts a restoration surface, is quite inhomogenous as well as the amount of chewing cycles per day, statistical investigations have been performed to determine cyclic loading circumstances. Braem *et al.* [57] proposed average human chewing stresses between 5 MPa and 20 MPa at a chewing frequency of approximately 2 Hz. Daily chewing performance is difficult to observe, since chewing duration, chewing stresses, chewing cycles, food consistency or bilaterally changes during mastication account for a wide range of data [58]. The number of occlusal contacts per day at medium chewing forces was estimated to range between 300 to 700 cycles.

In dentistry, the loss of material due to non-antagonistic contacts have been defined as occlusal contact free area (CFA) wear. Occlusal contact area (OCA) wear has been designated as material loss by direct interaction of an antagonist with the restorative material. The restorative materials are measured experimentally together with amalgam and relative wear rates are determined. The wear rates are normalized relative to amalgam since it is a clinically proved and successful standard material [12].

GIC exhibit a CFA wear five times higher than amalgam and three times higher than resin composite materials [59]. However, in spite of well suited surface wear characteristics, some restorations experience sudden failure due to mechanical fatigue. Failure mechanisms such as void nucleation, crack propagation and detachment of particles or sudden, subcritical failure are common features in wear and fatigue [7,60]. In contrast to initial fracture strengths, fatigue results in subcritical damage accumulation over time. Close to clinically relevant chewing forces of approximately 5 to 20 MPa, cyclic fatigue characteristics might be evaluated either with a “Wöhler” or a “Staircase” routine [57,61]. It has to be realized that the corrosive action of oral fluids, in cements as well as in ceramics, and even in polymers, may contribute significantly to the crack growth sensitivity of a material [62]. Cyclic fatigue experiments clearly show a detoriation of restorative materials and account for a limited clinical lifetime. GIC, in contrast to resinous filling materials, improve their strength level over time due to water sorption and thus counteract a worse fatigue degradation. After one month of water storage, the strength level of the initial fracture strength is obtained, even under cyclic loading conditions [41].

### 4.3. Thermal Compatibility

The tooth structure and restorative materials in the mouth will expand upon heating by hot foods and beverages but will contract when exposed to cold substances. Such expansions and contractions may break the marginal seal of an inlay or other fillings in the tooth, particularly if the difference in coefficient of thermal expansion (CTE) is great between the tooth and the restorative material. Table 2 shows representative literature values of linear CTEs for a selection of dental restorative materials in comparison with human enamel and dentin [18,63]. Within a practically relevant temperature range between 20 °C and 60 °C, materials such as resinous composites and amalgam expand more than the tooth tissue, whereas porcelain and glass ionomer cements are well adapted to the tooth tissue. Thermal mismatch of restorative materials and human tooth structure leads to thermal induced stressing of the cavity walls and, over time, to loss of marginal adaptation.

**Table 2:** Linear coefficients of thermal expansion of dental restoratives measured between 20 °C and 60 °C.

Material	CTE [ppm]
GIC	10.2–11.4
Resin composite	14–50
Amalgam	22.1–28.0
Porcelain	12.0
Human enamel	11.4
Human dentin	8.3

### 4.4. Adhesion to Tooth Structure

The chemical adhesion of GIC to enamel and dentin is achieved by reaction of phosphate ions in the dental tissue with carboxylate groups from the polyacrylic acid. Electro-neutrality is maintained by the displacement of calcium ions with the phosphate ions [64]. The glass-ionomer cements bond to dentin with values of tensile bond strength reported between 1 and 3 MPa [18]. These low values were observed due to the sensitivity of GIC to moisture during setting. The bond strength has been improved to 11 MPa by treatment of the dentin with a polycarboxylic acid cleaning agent [7,10]. Chemical adhesion of GIC to the hard tissue of teeth through the combination of polycarboxylic acids with hydroxyapatite has been cited as the most important advantage of the GIC. The ionic bonding mechanism between the acid and the hydroxyapatite is supported by observations that bond strength to enamel is greater than those to dentin, in correspondence with the relative amounts of hydroxyapatite in the two dental hard tissues [65]. It has been proposed that bonding results in polyacrylate ions replacing phosphate ions in the surface structure of hydroxyapatite. Although the exact mechanism is still unknown, it seems likely that it involves good wetting of the GIC and the subsequent formation of ionic bonds [66]. GIC bond directly to dentin and enamel, even in the presence of a smear layer. However, surface conditioners, such as polycarboxylic, citric or phosphoric acids, have been found to improve bond strength [67]. The conditioner acts as an etching agent which removes the smear layer from the dentin tubuli. The acids demineralise and penetrate a dentin surface layer to a depth of approximately 1 µm [68] and prepare for a chemical bonding.

### 4.5. Anticariogenic Properties

It is common knowledge that fluoride is the most effective agent in caries prevention [69]. Fluorides may act in different ways: The metabolism of the bacteria that cause caries is inhibited and the resistance of enamel and dentin is increased due to the remineralization of porous or softened enamel and dentin. Usually fluoride is applied as a solution, paste or varnish covering the whole dentition. The clinical experience of the anticariogenic effect indicates the benefits from fluoride releasing restorative materials [70]. However, a sustained, long-term fluoride release especially in marginal gaps between filling material and tooth help prevent secondary caries of the dental tissues [14]. For conventional GIC, an initial release of up to 10 ppm and a constant long-term release of 1 to 3 ppm over 100 months was reported [70]. This release (measured *in vitro* in distilled water) was evidenced to be capable of secondary caries prevention. In contrast, resin composite and compomer materials exhibit a reduced release between 0 and 1 ppm within the first seven days of water storage [71].

### 4.6. Clinical Performance

Fatigue fractures after several years of clinical service are a common cause of failure. Damage to restorations, such as bulk, cusp, or marginal fractures, were observed frequently [72,73]. Using resin composite materials, Burke *et al.* [74] reported marginal fracture (18%) and bulk fracture (7%) as the most prevalent reasons for re-restoration. Hickel *et al.* [13] reviewed annual failure rates in posterior stress-bearing cavities from literature findings. They determined median annual failure rates of 0–9% for resin-based composites, 0–7% for amalgam and 1.9–14.4% for GIC. They stated fractures as a main reason for failure. Another prospective clinical trial investigated a GIC in load-bearing class I and class II cavities. The study had to be abandoned after two years since 10% of the fillings had been fractured [75].

## 5. Reinforcing Concepts

### 5.1. Porosity Reduction

**Figure 5:**
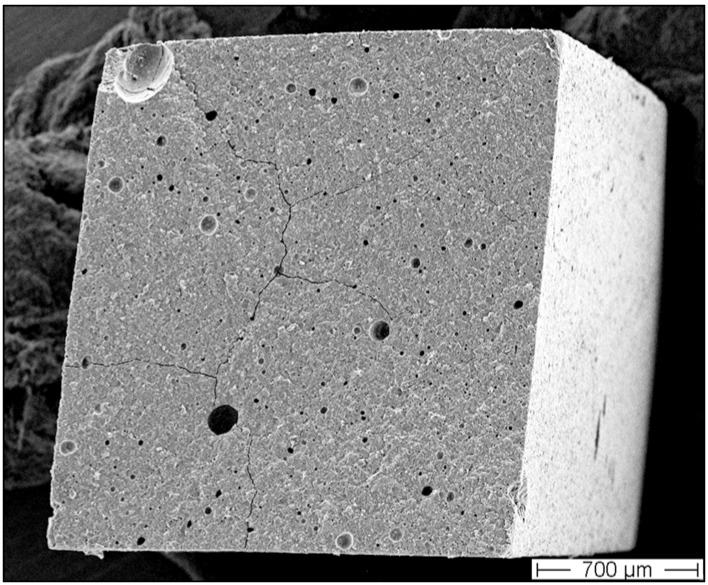
Typical pore size distribution in a commercial encapsulated GIC.

The mechanical properties of GIC are closely related to their microstructure. Factors such as particle size, or the distribution of porosity, affect the resulting strength significantly [11,76]. Variations in glass and liquid composition or powder/liquid ratio, glass particle size and pre-treatment as well as practical concerns like mixing by hand or in respective vibrational or rotational devices have a further influence on the final mechanical GIC properties [43]. Especially mixing is a concern of key importance, since any applied method is related to air entrapment into the cement structure (Figure 5). The amount and size of intrinsic porosity is reported to have a significant influence on mechanical properties [11,52]. A porosity of approximately 3.5% was found for hand mixed cements [77]. However, a reduced cement viscosity resulted in increased porosity [78]. Depending on the GIC viscosity, Nomoto *et al.* found a 10% decrease in strength at 0.2% porosity in a restorative GIC or even a 50% strength decrease in a 3% porosity containing luting cement [79].

In the daily practice it is difficult to ensure correct hand mixing and thus, pre-dosed capsules are provided to clinicians. Automatically mixed encapsulated cements ensure easy handling, a standardized and high p/l ratio, and a homogenous consistency to the cement paste. On the other hand a high level of porosity is incorporated in the final mix [80]. Trials comparing simple mixing in one axis with mixing and additional centrifugation resulted in more but smaller voids with the latter mixing procedure [81]. A reduced porosity of about one third and therefore an increased strength of 39% was found from mixing under vacuum [80].

### 5.2. Second Phase Particle Reinforcement

One idea to increasing cement strength and toughness was to incorporate metallic particles into the GIC matrix. A mixture (1:1) of conventional AgSn amalgam and the GIC glass particles was common in clinical use of the early days. The polycarboxylic acid when mixed with the powder, forms a plastic paste that progressively hardens with time [81]. A recent clinical study has shown that the durability of the so called “Cermets” [(Ceramic/metal) cements] as posterior restorative was inferior to conventional GIC [82,83]. The metal−matrix interfacial bonding of Cermets was lacking [84]. Obviously, the metallic appearance of Cermets accounts for their reduced esthetics. It has also been shown, that fluoride release is inferior compared to conventional GIC [85]. Hence, only little clinical value is attributed to metal reinforced cements.

The incorporation of short fibers was another promising attempt to achieve superior mechanical performance. A promising behavior was observed by compounding alumina, carbon, silicon nitride or E-glass fibers [14]. Kobayashi *et al.* started research into reactive short glass fiber reinforced glass-ionomer cements (FRGIC) [15]. They measured flexural strength 4.5 times higher than comparable unreinforced GIC. A 140% in flexural strength was measured by compounding 40 wt % reactive fibers [86]. However, the main advantage of fiber reinforcement is based on an increase in fracture toughness and work-of-fracture. Xu *et al.* [87] reported a 100-fold improvement in work-of-fracture and fourfold improvement in fracture strength by compounding short carbon fibers. Using reactive (ionomer) glass fibers, an increase in fracture toughness of 140% and of total energy release rate of 440% was measured, compared to the unreinforced GIC [88]. Matrix-fibre interface reaction is supposed to exert the major influence on mechanical behavior of FRGIC by controlling fibre pull-out and thus the total energy release rate.

The lack of sufficient release of incorporated bioactive agents has resulted in development of GIC for biomedical applications such as hard tissue replacement in the field of otological, oral-maxillofacial and orthopedic surgery. In order to increase bonding to bone, hydroxyapatite reinforced glass ionomer cements (HA-GIC) have been developed [89]. Fully crystalline HA powder was added to the GIC powder with comparable mechanical performance to the unreinforced counterpart. Further addition of nano-sized zirconia fillers to the HA-GIC has led to a significant increase in modulus, strength and hardness by keeping an improved dissolution stability with increased soaking time [90].

### 5.3. Resin Modification

A different strengthening approach has been introduced in the 1980s. Resin-modified glass ionomer cements (RMGIC) were developed to replace conventional GIC. According to McLean *et al.* [91], those materials generally set via a dominant acid-base reaction and auxiliary photopolymerization. With the addition of hydrophilic resin monomers (2-hydroxyethylmethacrylate (HEMA)), about 4.5 wt % [92], and a photo-initiator, RMGIC are polymerized immediately after visible light irradiation. Compared with their conventional counterparts, RMGIC have been characterized as having a longer working time, a rapid set, improved esthetic appearance and translucency, and higher early strength [93,94]. Due to the snap resin crosslinking upon photopolymerization of RMGIC, a two to three times higher compressive strength was observed when compared with conventional GIC in the initial fragile setting stage of the first 24 h [7]. However, RMGIC retain some properties of conventional GIC. Additional resin monomer and supplementary photopolymerization have not significantly reduced the susceptibility of RMGIC to dehydration problems [95]. Thus, the maintenance of water balance in the modified cements is still of importance. Only a few studies have addressed the importance of surface protection for RMGIC. Ribeiro *et al.* [96] proved the effectiveness and benefits of surface protection for the prevention of discoloration in RMGIC. Another study by Miyazaki *et al.* reported the influence of surface coatings on the flexural properties of both conventional and resin-modified GIC [97]. Their results indicated that RMGIC should be protected from water leaching for at least 1 hour after cement mixing. In contrast, most manufacturers’ instructions indicate that RMGIC can be used with or without surface protection.

Clinically, RMGIC are used in similar indications to GIC. A rapid set makes them more attractive in patients with a low compliance like children. On the other hand, RMGIC are reported to be more prone to abrasive wear due to a weak filler-matrix coupling [98]. Especially the higher fluoride release is one major argument for the use of GIC in high caries risk patients [99]. A recent clinical review on RMGIC has attested a generally good retention in class V cavities, with an annual failure rate over 13 years reported as being under 3% [100].

### 5.4. Resin Coating

**Figure 6:**
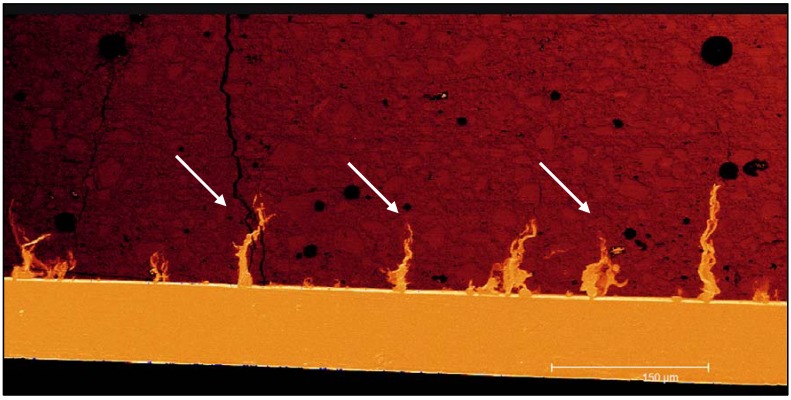
CLSM images of a resin coating layer on GIC. The complete sealing of surface porosities and dehydration cracks (arrows) is observable under fluorescent mode.

Since water plays a key role for proper maturation of a GIC, both water contamination and dehydration during the initial setting stages can compromise the physical properties of the restoration [43]. Gemalmaz *et al.* for example found in early moisture contaminated GIC restorations, that their mechanical strength dropped and their surface was prone to erosion and abrasion [101]. To prevent from those drawbacks, it is recommended to strictly exclude water during the vulnerable setting stage, which is reported to last for least one hour until even two weeks after placement [43]. Petroleum jelly, cocoa butter, waterproof varnishes, and even nail varnishes have been recommended in the past as suitable surface coating agents [102,103]. With time, those coatings are lost by oral masticative wear, but during this time, the cements become more resistant to variations in water balance due to their post-hardening [43]. Among the coating strategies, light-polymerized resin coatings have been considered the optimal surface protecting agent. Hotta *et al.* found, that the use of light-polymerized bonding or glazing agents are able to limit water movement across the setting cement surface [104]. Moreover, the ADA in 1990 declared the importance of varnishes or light-polymerized bonding agents for conventional GIC restorations [105]. Recently, a new restorative concept has been marketed (Equia^®^, GC Europe, Leuven, Belgium), a system application consisting of a posterior restorative GIC combined with a novel nanofilled coating material. This self-adhesive, nanofilled resin coating that provides a high hydrophilicity combined with an extremely low viscosity, accounts for a perfect seal of a GIC surface, as shown in Figure 6. Compounded nanofillers are thereby intended to protect the system against abrasive wear. This is of importance in the first months until the GIC is completely matured and able to withstand the intraoral stresses. The coating acts as a glaze, further increasing the esthetic properties [106]. Experimental studies have demonstrated the importance of controlling water loss in cements by the use of varnishes or other coatings. Not only crazing of the surface and loss of translucency can be avoided, but also strength may be affected [102]. Williams *et al.* found that when using metal-reinforced glass-ionomers, strength was significantly increased by coating the cements with varnishes or even with petroleum jelly [107]. Flexural strength has been determined for the system application Equia^®^ [108]. They showed a strength increase of 48% when comparing uncoated (16.8 MPa) with coated (32.2 MPa) specimens.

## 6. Outlook

GIC are favourable restorative materials due to their ease of use and unique biocompatibility among direct restoratives. However, brittleness limits their use in the load bearing posterior region. A low abrasion resistance and inferior strength, toughness and fatigue performance currently contraindicates the application as a permanent class I or class II filling materials. Several attempts in improving their mechanical parameters are still underway and some forecast a promising future for GIC as a dental filling material with extended indications.
